# Immune reconstitution inflammatory syndrome observed in the setting of drug-induced hypersensitivity syndrome/drug reaction with eosinophilia and systemic symptoms (DIHS/DRESS)

**DOI:** 10.1186/2045-7022-4-S3-P148

**Published:** 2014-07-18

**Authors:** Yoko Kano, Yukiko Ushigome, Chiho Horie, Yoshiko Mizukawa, Tetsuo Shiohara

**Affiliations:** 1 Kyorin University School of Medicine, Dermatology, Japan

## 

Immune reconstitution inflammatory syndrome (IRIS) is originally described in association with antiretroviral therapy (ART) for HIV infected patients. IRIS consists of a broad spectrum of inflammatory diseases, such as infectious inflammatory, neoplastic, and autoimmune diseases, that present after starting an effective ART, leading to CD4+ cells increase and plasma HIV-RNA reduction. IRIS reflects either worsening of an already-diagnosed infection or presentation of previously subclinical infection. Opportunistic pathogens include cryptococcus, mycobacterium, herpesviruses, or also auto-antigens. The paradoxical worsening of clinical symptoms as observed in IRIS is also the phenomenon of drug-induced hypersensitivity syndrome/drug reaction with eosinophilia and systemic symptoms (DIHS/DRESS). In the course of DIHS/DRESS, cytomegalovirus diseases and herpes zoster are observed coincidently with the increase in lymphocytes or rapid reduction of systemic corticosteroids. Based on the similar manifestations between IRIS and DIHS/DRESS, DIHS/DRESS can be seen in a broad context as another manifestation of IRIS. Although the mechanisms of IRIS is complex and variable, depending on the latent pathogens and shift of immune status, use of the concept of IRIS can help our recognition of various manifestations that occur in the setting of DIHS/DRESS. The understanding of IRIS may improve the morbidity and mortality rates of DIHS/DRESS.

